# Factors associated with presentation to care with advanced HIV disease in Brussels and Northern France: 1997-2007

**DOI:** 10.1186/1471-2334-11-11

**Published:** 2011-01-12

**Authors:** Bakhao Ndiaye, Julia Salleron, Anne Vincent, Pierre Bataille, Frédérique Bonnevie, Philippe Choisy, Karine Cochonat, Clotilde Fontier, Habib Guerroumi, Bernard Vandercam, Hugues Melliez, Yazdan Yazdanpanah

**Affiliations:** 1 CIRE Nord, Lille, France; 2 EA2694 Laboratoire de Biostatistique Faculté de Médecine de Lille, Lille, France; 3 Service Universitaire des Maladies Infectieuse Saint-Luc, Bruxelles, Belgique; 4 CH Boulogne, Boulogne, France; 5 CH Dunkerque, Dunkerque, France; 6 Service Universitaire des Maladies Infectieuses et du Voyageur, Tourcoing, France; 7 CH Lens, Lens, France; 8 CH Valenciennes, Valenciennes, France; 9 COREVIH Nord Pas-de-Calais, Tourcoing, France; 10 ATIP Avenir INSERM U995, Lille, France

## Abstract

**Background:**

Our objective was to determine the frequency and determinants of presentation to care with advanced HIV disease in patients who discover their HIV diagnosis at this stage as well as those with delayed presentation to care after HIV diagnosis in earlier stages.

**Methods:**

We collected data on 1,819 HIV-infected patients in Brussels (Belgium) and Northern France from January 1997 to December 2007. "Advanced HIV disease" was defined as CD4 count <200/mm^3 ^or clinically-defined AIDS at study inclusion and was stratified into two groups: (a) late testing, defined as presentation to care with advanced HIV disease and HIV diagnosis ≤6 months before initiation of HIV care; and (b) delayed presentation to care, defined as presentation to care with advanced HIV disease and HIV diagnosis >6 months before initiation of HIV care. We used multinomial logistic regression to determine the factors associated with delayed presentation to care and late testing.

**Results:**

Of the 570 patients initiating care with advanced HIV disease, 475 (83.3%) were tested late and 95 (16.7%) had delayed presentation to care. Risk factors for delayed presentation to care were: age 30-50 years, injection drug use, and follow-up in Brussels. Risk factors for late testing were: sub-Saharan African origin, male gender, and older age. HIV transmission through heterosexual contact was associated with an increased risk of both delayed presentation to care and late testing. Patients who initiated HIV care in 2003-2007 were less likely to have been tested late or to have a delayed presentation to care than patients who initiated care before 2003.

**Conclusion:**

A considerable proportion of HIV-infected patients present to care with advanced HIV disease. Late testing, rather than a delay in initiating care after earlier HIV testing, is the main determinant of presentation to care with advanced HIV disease. The factors associated with delay presentation to care differ from those associated with late testing. Different strategies should be developed to optimize early access to care in these two groups.

## Background

Despite the availability and known benefits of combined antiretroviral therapy (cART), a considerable proportion of HIV-infected patients have experienced significant immunodeficiency or disease progression before they present to care [1-7]. These patients often have poor prognoses [8], because cART reduces HIV RNA and mortality more effectively when it is initiated early [9-13]. In a recent study conducted in France, six-month mortality among patients who initiated care with advanced HIV disease, defined as CD4 count <200/mm^3 ^and/or AIDS, was 13.6 higher than among patients who initiated care early [2]. Mortality rates remained higher among these patients four years after enrolment. In addition to improving outcomes at the individual level, studies have shown that effective prevention counseling and early cART initiation can also reduce the clinical and financial burden of HIV at the population level by reducing HIV transmission rates [14-18]

HIV-infected patients who present to care with advanced HIV disease can be stratified into two groups: (i) patients who discover their HIV infection with an advanced HIV disease and thus initiate care late; and (ii) patients who discover their HIV infection earlier but delay initiating HIV care. Although several recent studies have evaluated the factors associated with presentation to care with advanced HIV disease[1-7], few studies have assessed the factors associated with presentation to care at this stage, by delay from first positive HIV test to presentation to care. The risk factors for presentation to care with advanced HIV disease may be different in these two groups.

The objective of this study was to determine the frequency and characteristics associated with late HIV testing with an advanced HIV disease and earlier HIV testing followed by delayed presentation to care with an advanced HIV disease among HIV-infected patients in Brussels (Belgium) and Northern France.

## Methods

### Patients

The Nord Pas-de-Calais region, one of 22 administrative regions in France, has a population of four million. Its cumulative incidence of AIDS is one of the lowest in France, at 299.4 cases per million inhabitants [19]. The Brussels administrative region is one of three in Belgium and has a population of one million. Its cumulative incidence of AIDS is one of the highest in Belgium, at 1,525 cases per million inhabitants [20].

We collected data on HIV-infected patients who received care at hospitals in Tourcoing, Valenciennes, Lens, Dunkerque, and Boulogne (Nord-Pas-de-Calais), as well as the Saint-Luc University Hospital in Brussels (Belgium) from January 1997 to December 2007. The Tourcoing AIDS Reference Centre of Lille University Teaching Hospital provides primary and subspecialty care to HIV-infected patients in the Lille-Roubaix-Tourcoing metropolitan area, the main urban area of the Nord Pas-de-Calais region. The Tourcoing AIDS Reference Centre has been described elsewhere in detail [21]. The hospitals in the smaller cities of Valenciennes, Lens, Dunkerque, and Boulogne also provide HIV care. Saint-Luc University Hospital [22] is one of two main hospitals that provide HIV in the Brussels metropolitan area.

Patients were eligible for the study if they received a confirmatory Western blot, were aged ≥18 years, had initiated care in one of the study centres, and gave informed consent to be included in the study. We considered that a patient had initiated care if he attended the study centers at least twice. We used standard questionnaires to collect complete prospective demographic, clinical, and laboratory data, as well as drug prescription information at the Tourcoing AIDS Reference Centre. Trained technicians in Tourcoing collected follow-up data at each outpatient visit and hospitalization, or at least every 6 months. Follow-up data were entered into standard computer records. In the other study centres, baseline and follow-up data were extracted retrospectively from patient charts, using the same standardized questionnaires as those used in Tourcoing.

### Definitions

Presentation with "advanced HIV disease" [7] was defined as initial presentation to care (i.e., the first clinical visit for HIV care) with CD4 count <200/mm^3 ^or clinically-defined AIDS. Presentation with "advanced HIV disease was stratified into two groups, according to the delay from the date of first positive HIV test result to the date of presentation to HIV care: (a) "late testing," defined as presentation with "advanced HIV disease" and HIV diagnosis ≤6 months before initial presentation to care; and (b) "delayed presentation to care," defined as presentation with "advanced HIV disease" and HIV diagnosis >6 months before initial presentation to care. In sensitivity analysis, we varied the CD4 count threshold at which patients presented to care with CD4 cell counts <50/mm^3 ^to 350/mm^3^.

### Variables

We assessed the factors associated with "late testing" and "delayed presentation to care" and compared them to the factors associated with "earlier presentation," defined as initiation of HIV care at CD4 counts >200/mm^3 ^and no AIDS.

We evaluated the demographic, clinical, biological and social characteristics of the patients included in the study. Demographic data included age at enrolment (<30 years, 30-39, 40-49 and >50), gender, origin (sub-Saharan African immigrant or not) and HIV transmission category. Gender and origin were stratified into four groups: non-immigrant women, non-immigrant men, immigrant men and immigrant women [2]. We stratified HIV transmission into four categories, in hierarchical order: injection drug users (IDU), men who have sex with men (MSM), heterosexual contact, and other. Patients who belonged to more than one transmission group were placed in the first relevant category of the hierarchy. AIDS-defining diseases at initial presentation to care were identified using the Expanded European AIDS definition [23]. We created three categories for period of inclusion: 1997-1999, 2000-2002, and 2003-2007.

### Statistical Analysis

We determined the proportion and characteristics of patients in the "delayed presentation to care" and "late testing" groups and compared them to patients who initiated care without advanced HIV disease. We used the chi 2 test to compare proportions and Wilcoxon's test to compare medians. In order to identify the factors that were independently associated with "delayed presentation to care" and "late testing," we entered the variables found to be associated with these outcomes in univariate analysis (p-value < 0.20) into a multinomial logistic regression model. In multivariate analysis, associations with p-values < 0.05 in Wald's test model were considered to be significant. We also evaluated the interaction between study country and the variables associated with "delayed presentation to care" and "late testing" in multivariate analysis. The significance level was set to 5%. All statistical analyses were performed using SPSS version 13.0 (SPSS Inc., Chicago. IL).

## Results

We collected data on 1,819 patients who were followed in Northern France and Brussels from January 1997 to December 2007. The initial characteristics of patients in Northern France and Brussels were the following: median age, 35 years (interquartile range [IQR], 28-43) and 36 years (IQR, 30-43; p = 0.07); proportion of men, 70.6% (860) and 56.2% (338; p < 0.0001); proportion of MSM, 44.9% (541) and 28.3% (156; p < 0.0001); proportion of IDU, 3.4% (41) and 1.8% (10; p < 0.0001); proportion of sub-Saharan African immigrants, 21.8% (265) and 53.1% (319; p < 0.0001); proportion of patients who initiated care in 2003-2007, 43.9% (535) and 54.9% (330), proportion of patients with AIDS-defining diseases at enrolment, 16.0% (195) and 11.1% (67; p = 0.005); and median CD4 count at inclusion, 353/mm^3 ^(IQR, 172-548) and 305/mm^3 ^(IQR, 165-485; p = 0.006) (Table 1).

**Table 1:** Demographic and clinical characteristics of HIV-infected patients in 6 clinical centres in Northern France and Brussels: January 1997 to December 2007

Characteristics	Northern France (n = 1218)	Brussels, Belgium (n = 601)	p-value
**Age, years**			
Median	35	36	0.07
25^th ^percentile	28	30	
75^th ^percentile	43	43	

**Gender**			<0.0001
Male	860 (70.6%)	338 (56.2%)	
Female	358 (29.4%)	263 (43.8%)	

**HIV transmission category**			<0.0001
Men who have sex with men	541 (44.9%)	156 (28.3%)	
Injection drug use	41 (3.4%)	10 (1.8%)	
Heterosexual contact	622 (51.7%)	385 (69.9%)	

**Sub-Saharan African immigrant**			<0.0001
Yes	265 (21.8%)	319 (53.1%)	
No	953 (78.2%)	282 (46.9%)	

**Period at enrolment**			<0.0001
1997-1999	337 (27.7%)	124 (20.6%)	
2000-2002	346 (28.4%)	147 (24.5%)	
2003-2007	535 (43.9%)	330 (54.9%)	

**AIDS-defining disease at enrolment**	195 (16.0%)	67 (11.1%)	0.005

**Baseline CD4 count,/mm**^**3**^			
Median	353	305	0.006
25^th ^percentile	172	165	
75^th ^percentile	548	485	

The proportion of patients who presented to care with advanced HIV disease was 31.3% and did not differ significantly between countries (Table 2). The numbers of patients who presented to care with AIDS or CD4 counts <50/mm^3^, and <350/mm^3 ^were 323 (17.6%), and 957(52.3%) (Table 2).

**Table 2:** The proportion of patients who presented to care with AIDS or CD4 counts <50/mm^3^, < 200/mm^3 ^and <350/mm^3 ^among HIV-infected patients in Northern France and Brussels: January 1997 to December 2007

		Prevalence		
**Presentation to care**	**Total** **n = 1819**	**Northern France** **n = 1218**	**Brussels** **n = 601**	**p-value**

CD4 count <50/μl or AIDS	323 (17.6%)	221 (18.1%)	102 (17.0%)	0.31
CD4 count <200/μl or AIDS*	**570 (31.3%)**	**373 (30.6%)**	**197 (32.8%)**	**0.18**
CD4 count <350/μl or AIDS	957 (52.9%)	614 (50.4%)	343 (57.1%)	0.21

*Advanced HIV disease

Table 3 shows the frequency of "late testing" and "delayed presentation to care" stratified by country." Nearly 17% of patients with advanced HIV disease were diagnosed earlier but delayed initiating HIV care and 83% were diagnosed late. Thirty nine of 95 (41.1%) patients with delayed presentation to care and 222 of 475 (46.7%) patients with late testing had an AIDS-defining illness at their initial presentation to care. Median time from HIV diagnosis to initial presentation to HIV care in the "delayed presentation to care" group was 55 months (IQR, 23-88). In this group, 85 patients (89.5%) tested positive for HIV >1 year before enrolling in the study. The frequency of "late testing" did not differ significantly between Northern France and Belgium, regardless of the definition of "late presentation" used. In contrast, the rate of "delayed presentation to care" did differ significantly between countries: "delayed presentation to care" was twice as frequent in Brussels than in Northern France (Table 3).

**Table 3:** Proportion of "delayed presentation to care" and "late testing" among HIV-infected patients who present to care late in Northern France and Brussels: January 1997 to December 2007

	Total n/N (%)	Northern France n/N (%)	Brussels, Belgium n/N (%)	p-value
Presentation to care with advanced HIV disease*and HIV diagnosis >6 months before initiation of care	95/570 (16.7)	44/373 (11.8)	51/197 (25.9)	0.0003

Presentation to care with advanced HIV disease*and HIV diagnosis ≤6 months before initiation of care	475/570 (83.3)	329/373 (88.2)	146/197 (74.1)	0.18

* CD4 < 200 cells/μl or AIDS

Table 4 shows the results of multinomial logistic regression analysis. The reference category was presentation to care without advanced HIV disease. Patients aged 30-40 years (odds ratio [OR], 3.28; 95% confidence interval [CI], 1.68-6.41) and 40-50 years (OR, 5.44; 95% CI, 2.69-11.02) were at higher risk of "delayed presentation to care" compared to patients aged <30 years. Compared to MSM, patients in the heterosexual contact transmission category (OR, 1.90; 95% CI, 1.14-3.21) and IDU (OR, 3.03; 95% CI, 1.14-9.53) were more likely to have tested earlier and presented to care with advanced HIV disease. Study inclusion in 1997-1999 was associated with "delayed presentation to care" compared to inclusion in 2003-2007 (OR, 3.20; 95% CI, 1.88-5.43). Patients in Brussels were also more likely to have tested early and presented to care with advanced HIV disease compared to patients in Northern France (OR, 2.34; 95% CI, 1.51-3.75).

**Table 4:** Multivariate multinomial logistic regression analysis: Factors associated with "delayed presentation to care" and "late testing" among HIV-infected patients in Northern France and Brussels, from January 1997 to December 2007.

	"delayed presentation to care" with advanced HIV disease* despite earlier HIV testing				"Late testing" and presentation to care with advanced HIV disease*			
	**n total**	**n (%)**	**OR**	**95% CI**	**n total**	**n (%)**	**OR**	**95% CI**

**Age, years**								
<30	454	12 (2.6)	1.00		529	87 (16.4)	1.00	
30-39	500	43 (8.6)	3.28	1.68-6.41	635	178 (28.0)	2.00	1.82-2.70
40-49	256	31 (12.1)	5.44	2.69-11.02	344	119 (34.6)	2.85	2.03-4.00
≥50	134	9 (6.7)	2.48	0.97-6.35	216	91 (42.1)	4.07	2.76-6.02

**Sex and immigrant status**								
Non-immigrant women					235	52 (22.1)	1.00	
Immigrant women					348	113 (32.5)	2.23	1.48-2.70
Non-immigrant men					980	254 (25.9)	1.86	1.23-2.80
Immigrant men					161	56 (34.8)	2.17	2.03-4.00

**HIV transmission category**								
Men who have sex with men	550	23 (4.2)	1.00		674	147 (21.8)	1.00	
Injection drug use	43	4 (9.3)	3.03	1.14-9.53	47	8 (17.0)	0.79	0.34-1.84
Heterosexual contact	709	63 (8.9)	1.90	1.14-3.21	944	298 (31.6)	1.56	1.13-2.14

**Study inclusion period**								
2003-2007	637	32 (4.8)	1.00		833	205 (24.6)	1.00	
2000-2002	332	26 (7.6)	1.76	0.99-3.14	467	151 (32.3)	1.58	1.21-2.06
1997-1999	333	37 (10.8)	3.20	1.88-5.43	424	119 (28.1)	1.42	1.07-1.88

**Country of follow-up**								
France	889	44 (4.9)	1.00					
Belgium	455	51 (11.2)	2.34	1.51-3.75				

Reference category is presentation to care without advanced HIV disease.

* CD4 < 200 cells/μl or AIDS

Rates of "late testing" increased as age increased from 30-40 years (OR, 2.00 compared to <30 years) to ≥50 years (OR, 4.07 compared to <30 years; p < 0.0001). Compared to non-immigrant women, immigrant women (OR, 2.23; 95% CI, 1.48-2.70), non-immigrant men (OR, 1.86; 95% CI, 1.23-2.80), and immigrant men (OR, 2.17; 95% CI, 2.03-4.00) were more likely to be diagnosed late. Compared to MSM, patients in the heterosexual contact transmission category were more likely to be diagnosed late (OR, 1.56; 95% CI, 1.13-2.14). Compared to patients who enrolled in the study in 2003-2007, patients who enrolled in 2000-2002 (OR, 1.58; 95% CI, 1.21-2.06) and 1997-1999 (OR, 1.42; 95% CI, 1.07-1.88) were more likely to have been diagnosed late.

## Discussion

We estimated the frequency of patients who presented to care with advanced HIV disease among 1,819 HIV-infected patients in the Nord-Pas-de-Calais region (France) and Brussels (Belgium) from January 1997 to December 2007. Moreover, within patients with advanced HIV disease we determined the frequency of "late testing" (i.e.; HIV diagnosis ≤6 months before initial presentation to care) and "delayed presentation to care" (i.e.; HIV diagnosis >6 months before initial presentation to care). Finally, we assessed the potential risk factors associated with "delayed presentation to care"/"late testing" and presentation to care with advanced HIV disease. Of the 570 patients (31.3%) who presented to care with advanced HIV disease, 95 (16.7%) were diagnosed early but delayed initiating HIV care for >6 months, and 475 (83.3%) were diagnosed late. Older patients, sub-Saharan African immigrants, non-immigrant men and patients who enrolled in the study between 1997 and 2002 were more likely to be diagnosed late. Risk factors for "delayed presentation to care" and presentation to care with advanced HIV disease were: age 30-49 years, HIV transmission through heterosexual contact or injection drug use, enrolment in the study between 1997 and 1999, and follow-up in Brussels.

There are multiple definitions of "late presentation" or "delayed HIV diagnosis" in the medical literature. Recently, The UK Collaborative HIV Cohort (UK CHIC) Steering Committee defined presentation at a stage when there is a substantial risk of death (i.e., CD4 counts <200/mm^3 ^or clinically-defined AIDS) as presentation with "advanced HIV disease" and presentation with a CD4 cell count below 350/mm^3^, resulting in a delay in treatment initiation, as "late presentation"[7]. In this analysis we focused on patients with advanced HIV diagnosis and showed that 31.3% of patients initiated care at CD4 counts <200/mm^3 ^or clinically-defined AIDS. This proportion was found to be similar in Northern France and Belgium, and is comparable to estimates from several recent European studies that used the same definition for "late presentation." In these studies, rates of "late presentation" ranged from 27% to 59% [1-7]. In our study, 16.7% of those with advanced HIV disease were aware of their HIV status but delayed initiating care for >6 months. Thus, late testing, rather than delay in initiating care after testing HIV positive, is the main determinant of presentation to care with advanced HIV disease. Of note, the proportion of patients who initiated care with an AIDS-defining event was comparable in patients with late testing and those with delayed presentation to care. This shows that late testing is also the main reason of presentation to care with AIDS. Few studies, especially in Europe, have focused their analysis on the group of patients delaying HIV care. In the Italian Cohort Naive Antiretrovirals, 26.1% of all HIV infected patients delayed initiating care for >6 months after HIV diagnosis, and these patients represented 26.1% of patients with <200 CD4 cells/mm^3 ^or clinically defined AIDS upon enrolment [24]. This estimate, using the same definition, is higher than the results from our study in Northern France (11.8% with advanced HIV disease had delayed initiating care) but close to those in the Belgium clinical cohort (25.9% with advanced HIV disease had delayed initiating care)

Consistent with previous studies, our findings show that older patients, heterosexuals, sub-Saharan African immigrants, non-immigrant men and patients who initiated care between 1997 and 2002 were more likely to be diagnosed late with advanced HIV disease [25-32]. The risk of "late testing" increased with age, from an OR of 2.00 among patients aged 30-40 years to 4.07 among patients aged ≥50 years, compared to patients aged <30 years. Previous studies have estimated that the risk of "late testing" in older age groups ranges from 3 to 6.5 [2,11,24,33-36]. A recent review evaluated the psychosocial factors associated with HIV testing in high-income countries. The authors concluded that individuals generally get tested for HIV for the first time when they perceive that they have been at risk [37]. Older individuals and heterosexuals may not feel at risk of HIV infection. Our finding that sub-Saharan African immigrants are at risk of presentation with advanced HIV disease corroborates previous reports that this group faces obstacles to HIV testing. A British study that investigated HIV testing practices among African immigrant communities found that although general HIV awareness was high, perception of individual risk was poor [38]. Furthermore, they found that cultural norms and stigma in immigrant communities often contributed to late presentation and remained a major barrier to HIV testing [11]. Immigrants usually present to care with immediate and specific needs, and the idea of getting tested for a disease before having symptoms, even when the risk of infection is high, is often rejected. Our finding that a large proportion of late HIV diagnoses were among non-immigrant men was also consistent with previous studies [2]. While women are systematically tested for HIV during pregnancy, men are not generally offered HIV tests on a routine basis.

Although several studies have investigated the factors associated with "late presentation" and "late testing," few studies have evaluated the characteristics of patients who get diagnosed with HIV early but delay presenting to care. We show that the risk factors for "delayed presentation to care" and "late testing" among patients who present with advanced HIV disease are different. First, we found that IDUs are more likely to test early and delay presenting to care. This result is consistent with previous studies [24] and demonstrates that IDU present with advanced HIV disease not because they are tested for HIV infrequently, but rather because they are not efficiently linked to care. In fact, IDU regularly receive HIV tests in specialized drug treatment centres. Moreover, because they are considered to be at high risk of HIV infection, physicians frequently offer them HIV tests during regular clinic visits. Despite their tendency to be diagnosed early, however, IDU are difficult to enroll and retain in care [21,25,39-43].

Second, although we found that rates of "late testing" increase with age, we did not find the same trend in the "delayed presentation to care" group. Patients aged 30-49 years were at increased risk of "delayed presentation to care" and presentation to care with advanced HIV disease compared to patients aged <30 years, but patients aged >50 years were not. Unlike patients aged >50 years, those aged 30-49 years may seek an HIV test because of a perceived risk, but then delay presenting to care to avoid HIV-related stigma. The number of patients >50 years in our cohort was small, however, so any interpretation of this result should be made with caution. Finally, we found that patients who were followed in Brussels were twice more likely to be diagnosed early and delay presenting to care than patients who were followed in Northern France. Of note, as stated above, the proportion of patients diagnosed early with presentation to care at advanced HIV disease in Brussels was close to estimates in the Italian Cohort Naive Antiretrovirals [24]. This finding may be related to different testing and counselling services and/or linkage to care services in different countries. Girardi et al. for example have shown in their study that having no counselling at the time of first positive test were associated with a higher probability of delayed presentation to care[24], but unfortunately this information was not available in our datasets. Differences may be also explained by differences in patient characteristics. Our findings may be for example explained by the higher proportion of sub-Saharan African immigrants in Brussels when compared to Northern France. In multinomial logistic regression analysis, we adjusted our results for patient characteristics, including patient origin, but we may not have adjusted our results for all potential confounders. Differences between our study and the Italian study maybe also related to difference in the time period during which the analysis was conducted. As seen in previous studies, in our study rates of "late testing" and "delayed presentation to care" decreased overtime [24,33]. This trend is likely related to the increasing availability of more effective and less toxic cART regimens, as well as improvements in HIV care.

Our study has several limitations. First, our results cannot be generalized to locations outside of Northern France and Brussels, because the risk factors associated with "delayed presentation to care" and "late testing" may depend on population characteristics and local HIV screening practices. Although most of our results are consistent with previous studies, some risk factors for "delayed presentation to care" and "late testing" may depend on geography: patients in Brussels were more likely to be diagnosed early and delay presentation care compared to patients in Northern France. Second, although most data were collected prospectively, the study was designed after data collection had ended. We were therefore unable to assess the impact of variables such as incarceration, homelessness, illegal immigrant status, unemployment status, HIV testing history, psychological condition, counselling and community support on outcomes. These variables might influence rates of "delayed presentation to care" and "late testing." Third, the delay from initial HIV diagnosis to initial presentation to care, which was used to differentiate patients in the "late testing" and "delayed presentation to care" groups, was often self-reported and thus subject to recall bias. Finally, we may not have had enough statistical power to evaluate some of the risk factors associated with "delayed presentation to care", because the number of patients who were diagnosed early and delayed presentation to care was small.

## Conclusion

Despite the wide availability of cART in Western Europe, access to care with advanced HIV disease remains common in France and Belgium, with almost one third of HIV-infected patients initiating care at CD4 counts <200/mm^3 ^and/or AIDS in recent years. This study shows that, although late HIV testing is the most frequent cause of delayed presentation to care, low rates of linkage to care also lead to late presentation. In our study, 17% of patients delayed initiating care after being diagnosed with HIV. Because the factors associated with "delayed presentation to care" differ from those associated with "late testing," different strategies should be developed to optimize early initiation of HIV care in each group. One method for increasing the proportion of patients who are diagnosed early is to broaden HIV screening practices. In France, the Haute Autorité de Santé [44,45] recently recommended one-time universal, routine, voluntary HIV screening in the general population, and more frequent screening in higher-risk groups. General practitioners in particular should be encouraged to provide HIV tests on a routine basis, in order to improve access to care at the individual level and limit the spread of infection at the population level [46,47]. Interventions to improve linkage to care should be implemented alongside HIV screening strategies, particularly in marginalized groups such as IDU, who tend to be diagnosed early but to delay presentation to care.

## Abbreviations

AIDS: Acquired immune deficiency syndrome; cART: combined antiretroviral therapy; CI: confidence interval; HIV: human immunodeficiency virus; IDU: injection drogue use; IQR: interquartile range; MSM: men who have sex with men; OR: odds ratio.

## Competing interests

YY has received honoraria for presentation at workshops and consultancy honoraria from Bristol-Myers Squibb, Gilead, Glaxo-SmithKline, Merck, Pfizer, Roche and Tibotec. In Brussels, Tibotec has helped the implementation of the database. Other authors declare that they have no competing interests.

## Authors' contributions

BN, JS, BV and YY were involved in the overall study design and protocol development. BN, AV, PB, FB, PC, KC, CF, HM and HG collected the data and participated to their interpretation. BN and JS performed the statistical analysis. BN, HM, BV and YY participated in writing the manuscript, which all authors have reviewed and approved.

## Pre-publication history

The pre-publication history for this paper can be accessed here:

http://www.biomedcentral.com/1471-2334/11/11/prepub
